# Food Security and Hygiene Practice among Adolescent Girls in Maiduguri Metropolitan Council, Borno State, Nigeria

**DOI:** 10.3390/foods9091265

**Published:** 2020-09-10

**Authors:** Ruth Charles Shapu, Suriani Ismail, Norliza Ahmad, Poh Ying Lim, Ibrahim Abubakar Njodi

**Affiliations:** 1 Department of Community Health, Faculty of Medicine and Health Science, University Putra Malaysia, Serdang 43400, Malaysia; ruthyshapu52@gmail.com (R.C.S.); lizaahmad@upm.edu.my (N.A.); pohying_my@upm.edu.my (P.Y.L.); 2 College of Nursing and Midwifery, Damboa Road, Maiduguri, 600252 Maiduguri, Nigeria; 3 Department of Physical and Health Education, University of Maiduguri, 600230 Maiduguri, Nigeria; ibrahimnjodi@gmail.com

**Keywords:** food security, hygiene practice, adolescent girls, KoBoCollect Toolbox, health

## Abstract

Inadequate food security contributes to poor health outcome for all, including adolescent girls. The study aims at determining the level of food security and hygiene among adolescent girls. A school-based cross-sectional study was carried out among adolescent girls aged 10 to 19 years old from six schools in Maiduguri Metropolitan Council, Borno State. The instrument for data collection was a questionnaire using KoBoCollect Toolbox between June and July 2019. Multivariable logistic regression was used to identify the predictors of food security and hygiene among respondents. A majority of the respondents (73.5%) were in a very low food security level. Three factors were significantly associated with food security level, these included schools (*p* = 0.007), age of mother (*p* = 0.004), and occupation of mother (*p* < 0.001). School (*p* = 0.003), age (father *p* = 0.017; mother; *p* = 0.012), hygiene (*p* = 0.005), and occupation of mother (*p* = 0.002) were predictors of food security. About (46.6%) of respondents had poor hygiene practice, school (*p* = 0.016) was significantly associated with hygiene practice. School (*p* = 0.019; and *p* = 0.005) and food security (*p* = 0.009) were predictors of hygiene practice. This study reveals a high prevalence of low food security among adolescent girls.

## 1. Introduction

Adolescence is a period of transition from childhood to adulthood, often divided into early (10–13 years), middle (14–16 years), and late (17–19 years) adolescent stages [1]. Globally an estimated 821 million people (one in every nine people) are malnourished as a result of food insecurity [2]. In developing countries, adolescents are exposed to environmental factors such as food insecurity and hygiene that may influence their growth, development, and well-being [3]. Everyone needs food to have appropriate nutritional status and also to sustain life [4]. Food insecurity and poor hygiene has become a problem among low-income households in developing and underdeveloped countries globally [5]. People do not have economic, social, and physical access to sufficient and nutritious food that can meet their dietary needs for an active and healthy life. Food must be available, accessible, utilized adequately, and in stable condition to meet nutritional demand [6].

Food security is a common problem among low-income households in developing and underdeveloped countries [5]. Food security is a priority, as malnutrition is a contributing factor to morbidity, mortality, reduced productivity, and poor cognitive development [7]. The global growth of population, the changes in climate, the rising price of food, and insurgency have a severe impact on food security [8]. In 2019 and 2020, the portion of the population facing food insecurity increased, and between 112 and 123 million people across 50 countries in the world were in crisis or in the worst form of food insecurity, with the Democratic Republic of the Congo and South Sudan reflecting acute food insecurity. The emergent severity of economic shocks and drought in countries such as Haiti, Pakistan, and Zimbabwe were found to be drivers of food insecurity. About 183 million people in 47 countries were classified in stressed food insecurity condition [9]. In 2019, the acute food insecurity level was almost 5 million (5%) of the population in 16 states analyzed with federal capital territory (FCT) inclusive in Nigeria. In Nigeria, in the three northeastern states affected by insecurity, about 3 million people from Borno (1.8 million), Yobe (945,000), and Adamawa state (297,000) were in an acute food insecurity level [9]. Food insecurity is a contributing factor to poor health outcomes and deficiency of nutrients among adolescents most likely to be due to inadequate intake of nutrients [10,11,12].

**Figure 1:**
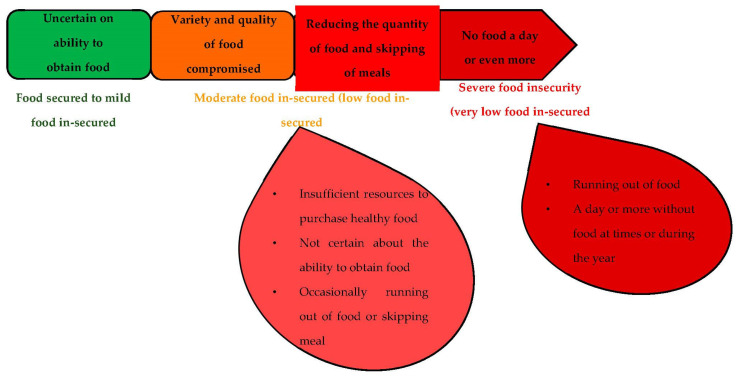
Food insecurity scale [2].

Food insecurity normally exists where there are limitations or uncertainty in the availability of adequate foods and in the ability to secure foods in an acceptable way [13,14]. About 37 million people from 11 African countries were declared food insecured in 2017 with the largest population from the north-eastern part of Nigeria, Congo, Somalia, and South Sudan. About 70% of the people of Borno were food insecured with some local governments still in the emergency phase, this has an impact on the internally displaced and host communities, especially households, that are entirely dependent on the market rather than on food production as shown in Figure 1 [6].

**Figure 2:**
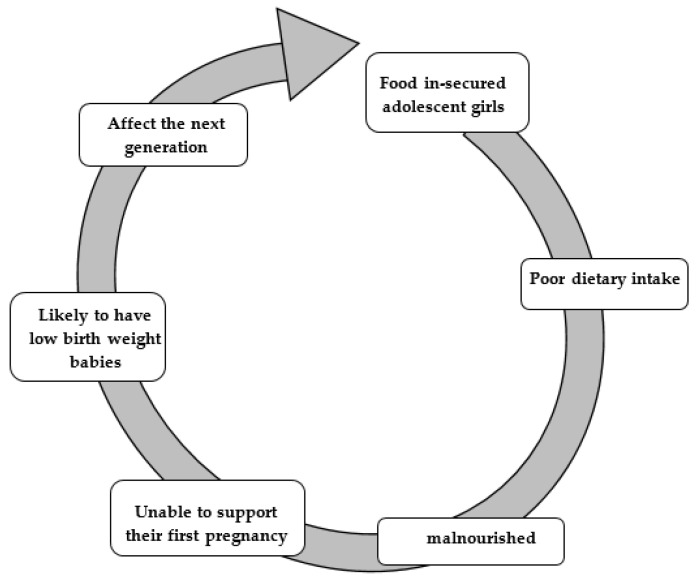
Negative consequences of food insecurity on adolescent girls.

The provision of food continuously has remained a serious challenge, as food is one of the basic needs of humanity for optimal nutrition [15]. In the low-income household, the burden of food insecurity is more among adolescents than in younger children [16]. Food insecurity has negative consequences and also affects all age groups, including adolescents. Adolescents girls from food insecured households tend to have lower academic performance and poor health and nutritional status [17,18,19,20]. Adolescent girls are at risk of becoming malnourished due to poor dietary intake. They are likely to have a low birth weight baby, which can impact the next generation as the prevalence of low birth weight babies is more among adolescent girls, as shown in Figure 2 [10,21,22].

Globally about 2.3 billion people do not have the essential sanitation services, nearly 892 million individuals are still practicing open defecation, and approximately 844 million people lack access to a good source of drinking water [23,24]. Lack of proper water facilities, water storage, water treatment, hand washing, and hygiene practices affects the health outcome of children under 18 years old. The long-term adverse effect of infection such as worm infestation, diarrhea, and dehydration attributed to poor water, sanitation, and hygiene conditions contributes to impaired learning ability among children under 18 years [25].

Assessing the magnitude of food security and hygiene practice of adolescent girls in the Northeast, this study will provide baseline data and the need for intervention. This study aims at determining the level of food security and hygiene among adolescent girls in Maiduguri Metropolitan Council, Borno State, Nigeria. 

## 2. Materials and Methods 

The details of the study design and methodology are explained in a research article titled “Knowledge, Attitude, and Practice of Adolescent Girls towards Reducing Malnutrition in Maiduguri Metropolitan Council, Borno State, Nigeria: Cross-Sectional Study” [26]. A brief information of the study design is given below.

This was a school-based cross-sectional study carried out among adolescent girls 10–19 years old in six secondary schools. The schools include Government Girls Secondary School Yerwa (Yerwa), Government Girls College Maiduguri (GGC), Government Girls Secondary School Maiduguri (GGSS), Shehu Garbai Day Secondary School (SGDSS), Bulabulin Day Secondary School (BDSS), and Zajeri Day Secondary school (ZDSS) in Maiduguri, Borno State, Nigeria. The main occupations of the state are farming/fishing and trading. In 2017, only 8.3% of the populace consumed from their crop and animal production, the remaining relied on purchased food due to increased displacement of the people from their own homes and farmland to places with limited farms for agricultural activities [27]. The sample size was 612; two-stage random sampling was used to select the school and the individual respondent.

A questionnaire through respondent’s interview using KoBoCollect Toolbox was used for data collection. KoBoCollect Toolbox is a data collection tool developed by Harvard, the application can run on any Android device either phone or tablet where data collected were stored in cloud storage together with the global positioning system (GPS) for the location of each school, and later exported to SPSS for analysis [28]. Information on sociodemographic characteristics was collected, details of sociodemographic characteristics were explained in a research article titled “Knowledge, Attitude and Practice of Adolescent Girls towards Reducing Malnutrition in Maiduguri Metropolitan Council, Borno State, Nigeria: Cross-Sectional Study” [26]. A food security questionnaire was adopted from food security for older children consisting of nine statements with options “a lot”, “sometimes”, and “never”. Never was scored “1”, sometimes “2”, and a lot “3” in the last 30 days [29]. Never was recoded “0”, sometimes and a lot weres recorded “1”. The total food security score was 9. Respondents who scored 0–1 were considered food secured, those with 2–5 were regarded as low food secured, and those with 6–9 were considered very low food secured, respectively [30]; these questions are shown in Table 1.

The hygiene section consists of five statements with options, choose one. Hygiene statements and characteristics are presented in the result section (Table 5). Frequency and percentage were used for each hygiene item. Scores <median were considered poor hygiene, while scores ≥median score were considered good hygiene.

Data analysis was done using Statistical Package for Social Sciences (SPSS) software version 25 used to solve research problems. Frequency and percentage were used for the distribution of each statement. Chi-square was used to determine the association of sociodemographic characteristics with food security and hygiene; multiple logistic regression was used to determine the predictors of hygiene, while multinomial logistic regression was used to determine the predictors of food security. Data with *p* < 0.25 in simple logistic regression were analyzed in multiple logistic regression. Variables with *p* < 0.05 were considered statistically significant in this study. 

The Ethical Committee for Research involving Human Subjects of Universiti Putra Malaysia (JKEUPM) UPM/TNCPI/RMC/JKEUPM/1.4.18.2 gave ethical approval, and permission for various schools was obtained from the Ministry of Education Maiduguri, Borno State, Nigeria. Pan African Clinical Trials Registry (PACTR201905528313816).

## 3. Results

A total of 612 respondents were included in the study, the respondents were randomly selected based on the inclusion criteria of the study. About 562 respondents consented and were interviewed. The response rate for this study was 92%. The detailed sociodemographic characteristics of respondents were explained in a research article titled “Knowledge, Attitude and Practice of Adolescent Girls towards Reducing Malnutrition in Maiduguri Metropolitan Council, Borno State, Nigeria: Cross-Sectional Study” [26].

### 3.1. Food Security

#### 3.1.1. Food Security Distribution of Respondents 

Table 1 shows the distribution of food security statements among respondents. The median, interquartile range (IQR) for food security was 8.0 (4.0). Almost three-quarters of the respondents did worry that food at home would run out before the family got money to buy more food sometimes (74.7%); in some cases, food bought ran out, and they did not have money to get more (69.9%); for others, their meals only included a few kinds of cheap foods because the family was running out of money to buy food (70.1%); some were not able to eat a balanced meal because the family did not have enough money (76.0); others ate less because the family did not have enough money to buy food (67.6); sometimes, the size of the meals was cut down because the family did not have enough money for food (62.6%); some skipped a meal because the family did not have enough money for food (63.0%); and others were hungry but did not eat because the family did not have enough food (63.0%). Table 2 reveals that a majority of respondents (73.5%) were in a very low food security level.

#### 3.1.2. Association and Predictors of Food Security among Respondents

##### Association between Sociodemographic Characteristics and Food Security

Table 3 shows the association of sociodemographic characteristics and hygiene with food security level. Three factors were significantly associated with food security level, these included schools (*p* = 0.007), age of mother (*p* = 0.004), occupation of mother (*p* < 0.001).

##### Predictors of Food Security among Respondents

Factors with *p* < 0.25 in simple multinomial logistic regression were tested in multiple multinomial logistic regression, including school, age of adolescent, place of residence, monthly income, head of household, age of the father, education of father, age of mother, education of mother, occupation of mother, family type, and hygiene.

Table 4 identified five factors that were significant predictors of food security. Respondents in BDSS compared to ZDSS were less likely to be in a low food security level compared to a very low food security level (Adjusted Odds Ratio (AOR) = 0.306, 95% confidence interval (CI): 0.140–0.671, *p* = 0.003). Respondents with age of father and mother between 35 and 44 years compared to ≥45 years were more likely to be in low food security level compared to very low food security level (AOR = 2.610, 95% CI: 1.187–5.736, *p* = 0.017; AOR = 2.180, 95% CI: 1.191–3.993, *p* = 0.012). Respondents with poor hygiene compared to good hygiene were more likely to be in low food security level compared to very low food security level (AOR = 1.979, 95% CI: 1.228–3.187, *p* = 0.005). Respondents with the age of mother between 35 and 44 years compared to ≥45 years were less likely to be in a food secured level compared to a very low food security level (AOR = 0.414, 95% CI: 0.187–0.917, *p* = 0.030) Respondents whose mothers were civil servants compared to housewives were more likely to be in a food secured level compared to a very low food security level (AOR = 4.144, 95% CI: 1.665–10.312, *p* = 0.002). 

### 3.2. Hygiene Practice

#### 3.2.1. Hygiene Distribution of Respondents 

Table 5 describes the hygiene of respondents. A high number of respondents (49.3%) reported borehole as their main source of water, (54.3%) used private latrine for defecating, (89.7%) answered correctly on hand-washing practice, (38.3%) used a clean, covered container to keep their drinking water, while (50.5%) of respondents used a clean cloth to strain their drinking water to make it safe for use at home. The median (IQR) for hygiene score was 10.0 (3.0). Poor hygiene was reported by (46.6%) of respondents.

#### 3.2.2. Association and Predictors of Hygiene among Respondents 

##### Association between Sociodemographic Characteristics and Hygiene

Table 6 showed association of sociodemographic characteristics and food security with hygiene level. School (*p* = 0.016) was significantly associated with hygiene.

##### Predictors of Hygiene among Respondents

Simple logistic regression was used to assess the association of sociodemographic characteristics, food security with hygiene. Factors with *p* < 0.25 in simple logistic regression were tested in multiple logistic regression, including school, age of adolescents, class, monthly income, education father, occupation father, education mother, occupation mother, family type, and food security level. 

Table 7 showed that two factors were statistically significant in predicting hygiene. Respondents in SGDSS and ZDSS were less likely to have good hygiene compared Yerwa (AOR = 0.472, 95% CI: 0.252–0.884, *p* = 0.019; AOR = 0.416, 95% CI: 0.227–0.762, *p* = 0.005). Respondents in a low food secured level were less likely to have good hygiene compared to those in a very low food secured level (AOR = 0.537, 95% CI: 0.337–0.855, *p* = 0.009).

## 4. Discussion

Food security is essential in achieving good health among adolescent girls, neglecting the aspect of food security may have implications for achieving sustainable development goals: goal 1 (eradicating poverty), goal 2 (ending hunger and all forms of malnutrition; attaining food security; promoting sustainable agriculture; addressing the nutritional needs of adolescent girls, pregnant, and lactating mothers), goal 3 (guarantee healthy lives and promote well-being for all at all ages) [31,32]. Food security and nutritional status influences the growth and development of adolescents as it becomes woven in an intergenerational cycle of malnutrition. Food insecurity deters adolescents from attaining a normal nutritional status, therefore, it is important that adolescent girls become well-nourished at all stages of growth and development. The effects of malnutrition in women are borne throughout their lifecycle and through generations. Nutritional inadequacy through food insecurity during the period of adolescence can affect their present and future health and well-being, as it is intrinsically linked to the health and well-being of their offspring [33,34].

In this study, 69.9% of respondents reported running out of food and not having enough money to get more, 76% not able to eat a balanced meal because the family did not have enough money, 67.6% ate less because there was no money to buy food, 62.6% cut their meals because there was not enough money for food, 63.0% were hungry but did not eat as shown in Table 1. The result in this study was higher than the study conducted in Jordan [35,36]. This may be due to non-availability of sufficient food and financial resources as a result of the displacement of the populace caused by insecurity, which has limited crop production and food availability. More so, low awareness on coping strategies could be a contributing factor.

This study showed that more than half of respondents (73.5%) were in a very low food security level presented in Table 2, this was higher than research conducted in Canada [37], Dhaka city [7], Southeast, Nigeria [12,38], Ethiopia [39], Boston [40], Ethiopia [41,42,43], Australia [44], and the U.S. [45]. The differences in the outcome of the study may be due to the humanitarian crisis experienced by the region from 2009 to date that has led to the displacement of people from their homes and farmlands. Families in studies with lower percentages of very low food secured level might be receiving sufficient government aid and more so differences in demographic characteristics could contribute to the small number of very low food secured level.

Age of mother was statistically associated with food security as described in Table 3. This was similar to a study conducted in Iran [46], food insecurity increases with increase in age, as the age of the mother increases, the number of children also increases as well as the size of the household, these can affect the quality and quantity of food consumed by households thereby increasing their risk of becoming food insecured [46]. Occupation of mother was significantly associated with food security. This is in line with studies in Korea [47] and Ecuador [48], although food insecurity is not merely determined by financial poverty, occupation of the mother increases household income, contributes to the household purchasing power, helps in diversifying their food, and improves their socioeconomic status, thereby enabling them to become food secured [46,49]. The occupation of the mother is an important determinant of food insecurity especially in low-income families [50].

In this study, occupation of the mother was a predictor of food security, this concurs with the study conducted in the Southeast, Nigeria [12] and Ethiopia [49]. Mother’s occupation was associated with food secured level compared to very low food secured level as mothers who earn a salary are better able to manage the household, thereby reducing the level of food insecurity among adolescent girls. Furthermore, this study hypothesizes that school, age of the father, age of the mother, and hygiene were significant predictors of food security among adolescent girls in Maiduguri Metropolitan Council, Borno State, as shown in Table 4.

A majority of respondents (49.3%) in Table 5 used a borehole as their main source of drinking water. The result obtained was higher than the result obtained from Oyo State, Nigeria [51], and lower than the result obtained from Dangila town, Ethiopia [52]. More than half of respondents (54.3%) used private latrine for defecating. This was lower than the study conducted in Tanzania [53]. A majority of respondents in this study (89.7%) answered correctly on hand-washing practice. The result is in line with the study conducted in Oyo State, Nigeria [51], Minch town, Ethiopia [54], Angolela, Ethiopia [55], Dangila town, Ethiopia [52], Southern Ethiopia [56], and higher than the result obtained from Ethiopia [52] and Klang valley, Malaysia [57]. These may be due to awareness on the need for good hygiene practice to mitigate bacterial infection as a result of the influx of people from the local government areas displaced by humanitarian crisis. In this study, about (6.9%) boiled their water before drinking. The findings from Ibadan were higher [51]. A majority of respondents (50.5%) used a clean cloth to strain their drinking water to make it safe at home. This was lower than the result obtained in Ibadan, Nigeria [51]. About (42.9) allow the dirt to settle at the bottom of the container. This was higher than the outcome from Ibadan [51]. Allowing water to settle down and the use of clean cloth to strain their drinking water were the common ways of treating water, with a lower rate of boiling water. This could be associated with low awareness on the effective ways of treating water before drinking.

Poor hygiene was reported by (46.6%) of respondents. This was higher than results obtained from Ethiopia [54] and India [58]. Poor hygiene practice in this study may be due to inadequate awareness about good sources of drinking water, proper disposal of waste, effective storage, and water treatment.

The study in Table 6 hypothesizes that school was significantly associated with hygiene practice, while school and food security level were significant predictors of hygiene among adolescent girls, as presented in Table 7.

### Limitation

The study was a self-report of food security and hygiene statements that might lead to recall bias.

## 5. Conclusions

Drawing conclusions from this study, there is need for creating awareness on food security to prevent adolescent girls from the negative impact of growth, development, and undernutrition. There is also need for further research on food security as a protective factor in the outcome of adolescent health. The findings of this study recommend food security and hygiene intervention targeting adolescents to address food security- and hygiene-related problems in Borno State. Further studies on predictors and consequences of food security and hygiene are recommended. Poverty alleviation initiatives should be integrated with food security programmes to promote availability, accessibility, and consumption of diverse food to reduce the multitude of consequences of undernutrition. This study provides a significant contribution to the food security status and hygiene practice among adolescent girls in Maiduguri Metropolitan Council, Borno State, Nigeria.

The outcome of this study reveals a high prevalence of very low food security, with almost half of the girls having poor hygiene practice. School, age of the father and mother, occupation of mother, and hygiene were found to be significant predictors of food security, while school and food security were found to be significant predictors of hygiene among adolescent girls. There is need for further research to evaluate the prevalence of food security among adolescent girls in the remaining government schools, private schools, and also those out of school within the state capital to be able to generate enough evidence for policymakers. There should be a health education intervention study that is deeper and more practical in addressing the factors hindering adolescent girls from good hygiene practice. Schools should provide an access point to hand washing material to be monitored regularly.

## Figures and Tables

**Table 1:** Food security distribution among respondents (n = 562).

Food Security Statement	Never		Sometimes		A lot	
	n	%	n	%	n	%
Did you worry that food at home would run out before your family got money to buy more?	88	15.7	420	74.7	54	9.6
Did the food that your family bought run out, and you did not have money to get more?	119	21.2	393	69.9	50	8.9
Did your meals only include a few kinds of cheap foods because your family was running out of money to buy food?	65	11.6	394	70.1	103	18.3
How often were you not able to eat a balanced meal because your family did not have enough money?	68	12.1	427	76.0	67	11.9
Did you have to eat less because your family did not have enough money to buy food?	125	22.2	380	67.6	57	10.1
Has the size of your meals been cut because your family did not have enough money for food?	157	27.9	352	62.6	53	9.4
Did you have to skip a meal because your family did not have enough money for food?	162	28.8	354	63.0	46	8.2
Were you hungry but didn’t eat because your family did not have enough food?	160	28.5	354	63.0	48	8.5
Did you not eat for a whole day because your family did not have enough money for food?	247	44.0	276	49.1	39	6.9

**Table 2:** Food security level of respondents (n = 562).

Food Security Level	Frequency (n)	Percentage (%)
Very low food security level	413	73.5
Low food security level	108	19.2
Food secured level	41	7.3

**Table 3:** Factors associated with food security among respondents (n = 562).

Variables	Very Low Food Security n (%)/Mean ± SD	Low Food Security n (%)/Mean ± SD	Food Secured n (%)/Mean ± SD	X^2^/t	*p*-Value
**Name of school**					
Yerwa	76 (18.4)	24 (22.2)	8 (19.5)	24.287 ^a^	0.007 *
GGC	55 (13.3)	19 (17.9)	12 (29.3)		
GGSS	46 (11.1)	14 (13.0)	3 (7.3)		
SGDSS	51 (12.3)	13 (12.0)	8 (19.5)		
BDSS	128 (31.0)	16 (14.8)	5 (12.2)		
ZDSS	57 (13.8)	22 (20.4)	5 (12.2)		
**Age of Adolescent Girls (years)**				4.385 ^a^	0.356
Early adolescents	69 (16.7)	25 (23.1)	6 (14.6)		
Middle adolescents	213 (51.6)	45 (41.7)	22 (53.7)		
Late adolescents	131(31.7)	38 (35.2)	13 (31.7)		
**Class**				1.534 ^a^	0.957
JSS1	94 (22.8)	27 (25.0)	11 (26.8)		
JSS2	80 (19.4)	21 (19.4)	9 (22.0)		
SS1	113 (27.4)	25 (23.1)	9 (22.0)		
SS2	126 (30.5)	35 (32.4)	12 (29.3)		
**Ethnicity**				10.273 ^a^	0.852
Bura	33 (8.0)	9 (8.3)	6 (14.6)		
Kanuri	133 (32.2)	30 (27.8)	10 (24.4		
Hausa	41 (9.9)	12 (11.1)	5 (12.2)		
Marghi	41 (9.9)	8 (7.4)	3 (7.3)		
Shuwa	21 (5.1)	5 (4.6)	2 (4.9)		
Fulani	40 (9.7)	8 (7.4)	4 (9.8)		
Chibok	16 (3.9)	2 (1.9)	1 (2.4)		
Gwoza	56 (13.6)	24 (22.2)	7 (17.1)		
Other ethnic groups	32(7.7)	10 (9.3)	3 (7.3)		
**Religion**				0.721 ^a^	0.697
Christianity	88 (21.3)	20 (18.5)	7 (17.1)		
Islam	325 (78.7)	88 (81.5)	34 (82.9)		
**Place of residence**				3.529 ^a^	0.171
Rural	50 (12.1)	20 (18.5)	4 (9.8)		
Urban	363 (87.9)	88 (81.5	37 (90.2)		
**Household size**				1.067 ^a^	0.899
≤5 members	34 (8.2)	9 (8.3)	3 (7.3)		
6–8 members	151 (36.6)	43 (39.8)	13 (31.7)		
≥9 members	228 (55.2)	56 (51.9)	25 (61.0)		
**Monthly income**				4.777 ^a^	0.573
Less than ₦18,000	116 (28.1)	31 (28.7)	16 (39.0)		
₦18,000–₦30,000	143 (34.6)	41 (38.0)	16 (39.0)		
₦31,000–₦50,000	107 (25.9)	25 (23.1)	7 (17.1)		
₦51,000 and above	47 (11.4)	11 (10.2)	2 (4.9)		
**Head of household**				-	0.330
Father	375 (90.8)	96 (88.9)	39 (95.1)		
Mother	25 (6.1)	6 (5.6)	0 (0.0)		
Relations	13 (3.1)	6 (5.6)	2 (4.9)		
**Age group of father (years)**				3.846 ^a^	0.146
35 to 44	33 (8.3)	15 (14.7)	4 (10.0)		
≥45	365 (91.7)	87 (85.3)	36 (90.0)		
**Education of father**				13.237 ^a^	0.104
No education	38 (9.3)	17 (16.0)	2 (4.9)		
Informal education	60 (14.7)	12 (11.3)	10 (24.4)		
Primary education	13 (3.2)	1 (0.9)	2 (4.9)		
Secondary education	137 (33.7)	43 (40.6)	13 (31.7)		
Tertiary education	159 (39.1)	33 (31.1)	14 (34.1)		
**Occupation of fathers**				-	0.942
Civil service	150 (37.2)	33 (32.4)	13 (31.7)		
Trading/business	207 (51.4)	58 (56.9)	23 (56.1)		
Farming	31 (7.7)	7 (6.9)	3 (7.3)		
Other occupation ^c^	15 (3.7)	4 (3.9)	2 (4.9)		
**Age group of mother (years)**				15.495 ^a^	0.004 *
≤34	76 (18.6)	16 (15.2)	8 (20.00)		
35 to 44	199 (48.8)	68 (64.8)	13 (32.5)		
≥45	133 (32.6)	21 (20.0)	19 (47.5)		
**Education of mothers**				8.711 ^a^	0.367
No education	72 (17.6)	26 (24.5)	3 (7.3)		
Informal education	89 (21.7)	22 (20.8)	12 (29.3)		
Primary education	40 (9.8)	12 (11.3)	6 (14.6)		
Secondary education	140 (34.1)	32 (30.2)	15 (36.6)		
Tertiary education	69 (16.8)	14 (13.2)	5 (12.2)		
**Occupation of mothers**				26.140 ^a^	<0.001 *
Civil service	51 (12.7)	19 (18.1)	16 (41.0)		
Trading/business	171 (42.4)	50 (47.6)	11 (28.2)		
Farming	23 (5.7)	2 (1.9)	2 (5.1)		
Housewives	158 (39.2)	234 (32.4)	10 (25.6)		
**Family type**				6.746 ^a^	0.150
Monogamy	218 (52.8)	47 (43.5)	16 (39.0)		
Polygamy	166 (40.2)	50 (46.3)	23 (56.1)		
single parenting	29 (7.0)	11 (10.2)	2 (4.9)		
**Hygiene**				5.811 ^a^	0.055
Poor hygiene	180 (43.6)	60 (55.6)	22 (53.7)		
Good hygiene	233 (56.4)	48 (44.4)	19 (46.3)		

Yerwa: Government Girls Secondary School Yerwa; GGC: Government Girls College, Maiduguri; GGSS: Government Girls Secondary School, Maiduguri; SGDSS: Shehu Garbai Day Secondary School; BDSS: Bulabulin Day Secondary School; ZDSS: Zajeri Day Secondary School * significant (*p* < 0.05), ^a^ chi-square, JSS1: Junior secondary school 1; JSS2: Junior secondary school 2; SS1; Senior secondary 1; SS2: Senior secondary 2, other occupations ^c^: Malami (voluntary Quranic teacher).

**Table 4:** Multinomial logistic regression for predictors of food security.

Variables	Low Food Secured						Food Secured					
	B	Crude Odds Ratio	SE	95% CI		*p*-Value	B	Adjusted Odds Ratio	SE	95% CI		*p*-Value
				Lower Bound	Upper Bound					Lower Bound	Upper Bound	
**Very low food secured**	**Ref**						**Ref**					
**Intercept**	−2.181		0.419			<0.001	−2.401		0.620			<0.001
**Name of school**												
Yerwa	−0.130	0.878	0.382	0.416	1.856	0.734	−0.136	0.873	0.666	0.236	3.222	0.838
GGC	−0.207	0.813	0.409	0.365	1.811	0.612	0.591	1.806	0.605	0.552	5.908	0.328
GGSS	−0.356	0.701	0.455	0.287	1.710	0.435	−0.450	0.638	0.775	0.140	2.910	0.561
SGDSS	−0.375	0.688	0.426	0.299	1.583	0.379	0.165	1.179	0.643	0.335	4.156	0.797
BDSS	−1.184	0.306	0.400	0.140	0.671	0.003 *	−0.852	0.427	0.670	0.115	1.586	0.204
ZDSS	Ref						Ref					
**Age group of father (years)**												
35 to 44	0.959	2.610	0.402	1.187	5.736	0.017 *	0.531	1.700	0.667	0.460	6.288	0.427
≥45	Ref						Ref					
**Age group of mother (years)**												
≤34	0.123	1.131	0.424	0.493	2.597	0.771	−0.424	0.654	0.528	0.232	1.842	0.422
35 to 44	0.779	2.180	0.309	1.191	3.993	0.012 *	−0.883	0.414	0.406	0.187	0.917	0.030 *
≥45	Ref						Ref					
**Occupation of mothers**												
Civil service	0.544	1.722	0.367	0.839	3.534	0.138	1.422	4.144	0.465	1.665	10.312	0.002 *
Trading/business	0.495	1.640	0.274	0.960	2.804	0.070	0.089	1.093	0.460	0.443	2.696	0.846
Farming	−0.860	0.423	0.790	0.090	1.989	0.276	0.245	1.278	0.849	0.242	6.750	0.773
Housewives	Ref						Ref					
**Hygiene**												
Poor hygiene	0.682	1.979	0.243	1.228	3.187	0.005 *	0.418	1.519	0.363	0.746	3.093	0.249
Good knowledge	Ref						Ref					

Yerwa: Government Girls Secondary School Yerwa; GGC: Government Girls College, Maiduguri; GGSS: Government Girls Secondary School, Maiduguri; SGDSS: Shehu Garbai Day Secondary School; BDSS: Bulabulin Day Secondary School; ZDSS: Zajeri Day Secondary School. * Significant at *p* < 0.05, SE = standard error, CI = confidence interval, Ref = reference category, B = unstandardized beta, Cox and Snell R square = 0.126, Nagelkerke R square = 0.164, classification percentage = 74.1%, receiver operating characteristics = 75.5%, model fitting *p* < 0.001, goodness-of-fit Pearson *p* = 0.271, deviance *p* = 0.875.

**Table 5:** Hygiene distribution of respondents (n = 562).

Hygiene Statement	Characteristics	Frequency (n)	Percentage (%)
What is your current source of drinking water	Tap	93	16.5
	Borehole	277	49.3
	Well	12	2.1
	Dam	154	27.4
	Tanker	5	0.9
	Truck	21	3.7
Where do you dispose human waste (defecate) at home	Private latrine	305	54.3
	Shared latrine	237	42.2
	Open defaecation	20	3.6
What are the times when you wash your hands with soap or ash or sand and clean water	Correct answer	504	89.7
	Incorrect answer	58	10.3
I keep my water in a	Clean container	126	22.4
	Covered container	221	39.3
	A clean, covered container	215	38.3
In which of the following ways do you treat your drinking water to make it safe at home	Boil the water	39	6.9
	Use a clean cloth to strain it	284	50.5
	Allow the dirt to settle at the bottom of the container	239	42.5

**Table 6:** Factors associated with hygiene.

Variables	Level of Hygiene		X^2^/t	*p*-Value
	Poor n (%)	Good n (%)		
**Name of school**			14.017 ^a^	0.016 *
Yerwa	43 (16.4)	65 (21.7)		
GGC	34 (13.0)	52 (17.3)		
GGSS	27 (10.3)	36 (12.0)		
SGDSS	42 (16.0)	30 (10.0)		
BDSS	66 (25.2)	83 (27.7)		
ZDSS	50 (19.1)	34 (11.3)		
**Age of adolescent girls (years)**			2.663 ^a^	0.264
Early adolescents	54 (20.6)	46 (15.3)		
Middle adolescents	126 (48.1)	154 (51.3)		
Late adolescents	82 (31.3)	100 (33.3)		
**Class**			3.679 ^a^	0.298
JSS1	66 (25.2)	66 (22.0)		
JSS2	49 (18.7)	61 (20.3)		
SS1	75 (28.6)	72 (24.0)		
SS2	72 (27.5)	101 (33.7)		
**Ethnicity**			3.198 ^a^	0.921
Bura	22 (8.4)	26 (8.7)		
Kanuri	76 (29.0)	97 (32.3)		
Hausa	27 (10.3)	31 (10.3)		
Marghi	23 (8.8)	29 (9.7)		
Shuwa	12 (4.6)	16 (5.3)		
Fulani	23 (8.8)	29 (9.7)		
Chibok	10 (3.8)	9 (3.0)		
Gwoza	47 (17.9)	40 (13.3)		
Other tribes	22 (8.4)	23 (7.7)		
**Religion**			0.085 ^a^	0.771
Christianity	55 (21.0)	60 (20.0)		
Islam	207 (79.0)	240 (80.0)		
**Place of residence**			0.391 ^a^	0.532
Rural	37 (14.1)	37 (12.3)		
Urban	225 (85.9)	263 (87.7)		
**Household size**			0.269 ^a^	0.874
≤5 members	22 (8.4)	24 (8.0)		
6–8 members	99 (37.8)	108 (36.0)		
≥9 members	141 (53.8)	168 (56.0)		
**Monthly income**			2.181 ^a^	0.536
Less than ₦18,000	72 (27.5)	91 (30.3)		
₦18,000–₦30,000	97 (37.0)	103 (34.3)		
₦31,000–₦50,000	61 (23.3)	78 (26.0)		
₦51,000 and above	32 (12.2)	28 (9.3)		
**Variables**	Level of Hygiene	X^2^/t	*p*-Value	Variables
**Head of household**			0.159 ^a^	0.924
Father	238 (90.8)	272 (90.7)		
Mother	15 (5.7)	16 (5.3)		
Relations	9 (3.4)	12 (4.0)		
**Age group of father (years)**			0.421 ^a^	0.517
35 to 44	26 (10.5)	26 (8.9)		
≥45	221 (89.5)	267 (91.1)		
**Education of father**			3.481 ^a^	0.481
No education	28 (10.9)	29 (9.8)		
Informal education	45 (17.5)	37 (12.5)		
Primary education	7 (2.7)	9 (3.0)		
Secondary education	88 (34.2)	105 (35.4)		
Tertiary education	89 (34.6)	117 (39.4)		
**Occupation of fathers**			2.190 ^a^	0.534
Civil service	95 (37.8)	101 (34.2)		
Trading/business	126 (50.2)	162 (54.9)		
Farming	18 (7.2)	23 (7.8)		
Other occupations ^c^	12 (4.8)	9 (3.1)		
**Age group of mother (years)**			0.549 ^a^	0.760
≤34	43 (16.8)	57 (19.2)		
35 to 44	131 (51.2)	149 (50.2)		
≥45	82 (32.0)	91 (30.6)		
**Education of mothers**			2.334 ^a^	0.675
No education	49 (19.0)	52 (17.4)		
Informal education	59 (22.9)	64 (21.4)		
Primary education	25 (9.7)	33 (11.0)		
Secondary education	90 (34.9)	97 (32.4)		
Tertiary education	35 (13.6)	53 (17.7)		
**Occupation of mothers**			4.120 ^a^	0.249
Civil service	48 (18.5)	38 (13.2)		
Trading/business	102 (39.4)	130 (45.1)		
Farming	11 (4.2)	16 (5.6)		
Housewives	98 (37.8)	104 (36.1)		
**Family type**			2.032 ^a^	0.362
Monogamy	128 (48.9)	153 (51.0)		
Polygamy	110 (42.0)	129 (43.0)		
Single parenting	24 (9.2)	18 (6.0)		
**Food security**			5.811 ^a^	0.055
Very low food secured	180 (68.7)	233 (77.7)		
Low food secured	60 (22.9)	48 (16.0)		
Food secured	22 (8.4)	19 (6.3)		

* Significant at *p* < 0.05; ^a^ Chi-square; Yerwa: Government Girls Secondary School Yerwa; GGC: Government Girls College, Maiduguri; GGSS: Government Girls Secondary School, Maiduguri; SGDSS: Shehu Garbai Day Secondary School; BDSS: Bulabulin Day Secondary School; ZDSS: Zajeri Day Secondary School; other occupations ^c^: Malami (Voluntary Quranic teacher); JSS1: Junior secondary school 1; JSS2: Junior secondary school 2; SS1; Senior secondary 1; SS2: Senior secondary 2.

**Table 7:** Predictors of hygiene.

Variables	Simple Logistic Regression						Multiple Logistic Regression					
	B	Crude Odds Ratio	SE	95% CI		*p*-Value	B	Adjusted Odds Ratio	SE	95% CI		*p*-Value
				Lower Bound	Upper Bound					Lower Bound	Upper Bound	
**Name of school**												
Yerwa	Ref						Ref					
GGC	0.012	1.012	0.295	0.567	1.805	0.968	−0.021	0.979	0.312	0.531	1.806	0.947
GGSS	−0.126	0.882	0.322	0.470	1.657	0.696	−0.344	0.709	0.334	0.368	1.366	0.304
SGDSS	−0.750	0.473	0.309	0.258	0.867	0.015 *	−0.751	0.472	0.320	0.252	0.884	0.019 *
BDSS	−0.184	0.832	0.257	0.503	1.376	0.473	−0.331	0.718	0.273	0.420	1.227	0.226
ZDSS	−0.799	0.450	0.297	0.251	0.805	0.007 *	−0.876	0.416	0.309	0.227	0.762	0.005 *
**Age of adolescent girls (years)**												
Early adolescents	−0.359	0.699	0.250	0.428	1.140	0.151	-	-	-	-	-	-
Middle adolescents	0.002	1.002	0.191	0.689	1.458	0.991	-	-	-	-	-	-
Late adolescents	Ref						-	-	-	-	-	-
**Class**												
JSS1	−0.338	0.713	0.233	0.452	1.125	0.146	-	-	-	-	-	-
JSS2	−0.119	0.887	0.246	0.548	1.438	0.628	-	-	-	-	-	-
SS1	−0.379	0.684	0.226	0.440	1.065	0.093	-	-	-	-	-	-
SS2	Ref						-	-	-	-	-	-
**Ethnicity**												
Bura	Ref						-	-	-	-	-	-
Kanuri	0.077	1.080	0.328	0.568	2.053	0.814	-	-	-	-	-	-
Hausa	−0.029	0.972	0.391	0.451	2.092	0.941	-	-	-	-	-	-
Marghi	0.065	1.067	0.402	0.485	2/347	0.872	-	-	-	-	-	-
Shuwa	0.121	1.128	0.479	0.441	2.887	0.801	-	-	-	-	-	-
Fulani	0.065	1.067	0.402	0.485	2.347	0.872	-	-	-	-	-	-
Chibok	−0.272	0.762	0.543	0.263	2.208	0.616	-	-	-	-	-	-
Gwoza	−0.328	0.720	0.361	0.355	1.461	0.363	-	-	-	-	-	-
Other ethnic groups	−0.123	0.885	0.416	0.392	1.998	0.768	-	-	-	-	-	-
**Religion**												
Christianity	Ref						-	-	-	-	-	-
Islam	0.061	1.063	0.209	0.705	1.602	0.771	-	-	-	-	-	-
**Place of residence**												
Rural	Ref						-	-	-	-	-	-
Urban	0.156	1.169	0.250	0.717	1.906	0.532	-	-	-	-	-	-
**Household size**												
≤5 members	Ref						-	-	-	-	-	-
6–8 members	0.001	1.000	0.326	0.528	1.896	1.000	-	-	-	-	-	-
≥9 members	0.088	1.092	0.316	0.587	2.031	0.781	-	-	-	-	-	-
**Monthly income**												
Less than ₦18,000	Ref						-	-	-	-	-	-
₦18,000–₦30,000	−0.174	0.840	0.212	0.555	1.273	0.411	-	-	-	-	-	-
₦31,000–₦50,000	0.012	1.012	0.233	0.641	1.596	0.960	-	-	-	-	-	-
₦51,000 and above	−0.368	0.692	0.303	0.382	1.254	0.225	-	-	-	-	-	-
**Head of household**												
Father	Ref						-	-	-	-	-	-
Mother	−0.069	0.933	0.370	0.452	1.928	0.852	-	-	-	-	-	-
Relations	0.154	1.167	0.450	0.483	2.817	0.732	-	-	-	-	-	-
**Age group of father (years)**												
35 to 44	−0.189	0.828	0.292	0.467	1.467	0.517	-	-	-	-	-	-
≥45	Ref						-	-	-	-	-	-
**Education of father**												
No education	−0.238	0.788	0.300	0.438	1.418	0.427	-	-	-	-	-	-
Informal education	−0.469	0.625	0.263	0.374	1.047	0.074	-	-	-	-	-	-
Primary education	−0.022	0.978	0.523	0.351	2.727	0.966	-	-	-	-	-	-
Secondary education	−0.097	0.908	0.202	0.611	1.348	0.631	-	-	-	-	-	-
Tertiary education	Ref						-	-	-	-	-	-
**Occupation of fathers**												
Civil service	0.349	1.418	0.464	0.571	3.516	0.452	-	-	-	-	-	-
Trading/business	0.539	1.714	0.457	0.700	4.196	0.238	-	-	-	-	-	-
Farming	0.533	1.704	0.542	0.589	4.926	0.325	-	-	-	-	-	-
Other occupations ^c^	Ref						-	-	-	-	-	-
**Age group of mother (years)**												
≤34	0.178	1.194	0.253	0.728	1.961	0.482	-	-	-	-	-	-
35 to 44	0.025	1.025	0.194	0.701	1.498	0.899	-	-	-	-	-	-
≥45	Ref						-	-	-	-	-	-
**Education of mothers**												
No education	−0.356	0.701	0.295	0.393	1.250	0.228	-	-	-	-	-	-
Informal education	−0.334	0.716	0.283	0.411	1.247	0.238	-	-	-	-	-	-
Primary education	−0.137	0.872	0.343	0.445	1.708	0.689	-	-	-	-	-	-
Secondary education	−0.340	0.712	0.262	0.426	1.190	0.195	-	-	-	-	-	-
Tertiary education	Ref						-	-	-	-	-	-
**Occupation of mothers**												
Civil service	Ref						-	-	-	-	-	-
Trading/business	0.476	1.610	0.254	0.978	2.650	0.061	-	-	-	-	-	-
Farming	0.608	1.837	0.448	0.764	4.420	0.174	-	-	-	-	-	-
Housewives	0.293	1.340	0.259	0.807	2.226	0.257	-	-	-	-	-	-
**Family type**												
Monogamy	0.466	1.594	0.334	0.828	3.067	0.163	-	-	-	-	-	-
Polygamy	0.447	1.564	0.338	0.807	3.031	0.186	-	-	-	-	-	-
Single parenting	Ref						-	-	-	-	-	-
**Food security**												
Very low food secured	Ref						Ref					
Low food secured	−0.481	0.618	0.218	0.403	0.947	0.027 *	−0.622	0.537	0.237	0.337	0.855	0.009 *
Food secured	−0.405	0.667	0.329	0.350	1.270	0.218	−0.473	0.623	0.347	0.316	1.230	0.173
Constant							0.655	1.925	0.215			0.002

* Significant at *p* < 0.05, SE = standard error, CI = confidence interval, Ref = reference category, B = unstandardized beta, Cox and Snell R square = 0.083, Nagelkerke R square = 0.111, classification percentage = 58.7%, receiver operating characteristics = 59.8%, model fitting *p* = 0.003, Hosmer and Lemeshow *p* = 0.236, Yerwa: Government Girls Secondary School Yerwa; GGC: Government Girls College, Maiduguri; GGSS: Government Girls Secondary School, Maiduguri; SGDSS: Shehu Garbai Day Secondary School; BDSS: Bulabulin Day Secondary School; ZDSS: Zajeri Day Secondary School; Other occupation ^c^ = Malami (Voluntary Quranic teacher).
